# Pediatric Mortality in a Rural Tertiary Care Center in Liberia

**DOI:** 10.3390/children4020008

**Published:** 2017-01-30

**Authors:** Carmelle Tsai, Camila B. Walters, John Sampson, Francis Kateh, Mary P. Chang

**Affiliations:** 1 Department of Pediatrics, University of Texas at Southwestern, Dallas, TX 75390, USA; carmelle.tsai@gmail.com; 2 Department of Anesthesiology/Pediatric Anesthesiology, Vanderbilt University, Nashville, TN 37232, USA; camila.walters@vanderbilt.edu; 3 Department of Anesthesiology/Critical Care Medicine, Johns Hopkins University, Baltimore, MD 21205, USA; jsampso4@jhmi.edu; 4 Ministry of Health and Social Welfare, P. O. Box 10-9009 1000, Monrovia 10, Liberia; frankateh@aol.com; 5 Department of Emergency Medicine, University of Texas at Southwestern, Dallas, TX 75390, USA

**Keywords:** pediatrics, mortality, Liberia

## Abstract

Liberia is a low-income country in West Africa that has faced significant challenges, including a civil war and the recent Ebola epidemic. Little data exists on the more current post-war and pre-Ebola trends of child health in Liberia in the rural setting. This study is a retrospective chart review of pediatric mortality in 2013 at a rural tertiary care center in Liberia, 10 years post-war. From January 2013 to December 2013, there were 50 pediatric deaths, or 5.4% of the 920 total pediatric admissions. The most common cause of neonatal death was sepsis, and the most common cause of death under five years of age was malaria. The majority (82.0%) of the deaths were in children under five. Pediatric mortality at this hospital was similar to other reported mortality six years post-war, and lower than that reported immediately post-war. Neonatal sepsis and malaria are two significant causes of pediatric mortality in this community and, therefore, further efforts to decrease childhood mortality should focus on these causes.

## 1. Introduction

Liberia is a low-income West African country that has endured significant challenges. The multiple civil wars (1999 to 2003) and the recent Ebola crisis have affected the health care sector. In 1999, Liberia had the sixth highest under-five mortality rate (146 per 1000 live births) and the 15th highest neonatal mortality rate (46 per 1000 live births) in the world [1]. Since the cessation of war, Liberia has seen a marked improvement in its under-five mortality rate. In 2012, Liberia was one of six African countries to achieve the Millennium Development Goal 4 of reducing child mortality [1]. By 2015, Liberia’s under-five mortality rate dropped to 70 per 1000 live births, making it the 28th highest in the world, a significant feat for the country [2]. Despite these significant advances, pediatric health was likely significantly affected by the civil war. For example, the measles vaccination rate reached a nadir of 41% in 2004, immediately post-war. In response, significant post-war efforts to improve the public health of children led to a vaccination rate as high as 80% by 2012 [3]. Despite the impact of the civil war on child health in Liberia, there is a paucity of published data on the trends and current status of pediatric mortality in the post-war era. When the country was undergoing a crisis or war, the already-limited resources were diverted by priorities. With the recent Ebola crisis, many health care resources in the capital were diverted to Ebola treatment and hospitals and personnel were focused on monitoring the acute phase situation.

As Liberia works to accomplish the United Nations Sustainable Development Goals, it will be crucial to continue studying the complexities of child health and monitoring causes of pediatric mortality to inform health policy and governmental efforts. The goal of this study is to describe the 2013 pediatric mortality of a rural tertiary hospital in northern Liberia in the post-war, immediate pre-Ebola period to add to the existing body of literature.

## 2. Results

### 2.1. Overview

There were a total of 50 pediatric deaths in 2013, which was 5.4% of the 920 total patients admitted to the pediatric ward. The median age of death was one year of age. The most common reasons for admission in the pediatric ward were malaria, anemia, and pneumonia.

### 2.2. Causes of Mortality

**Figure 1:**
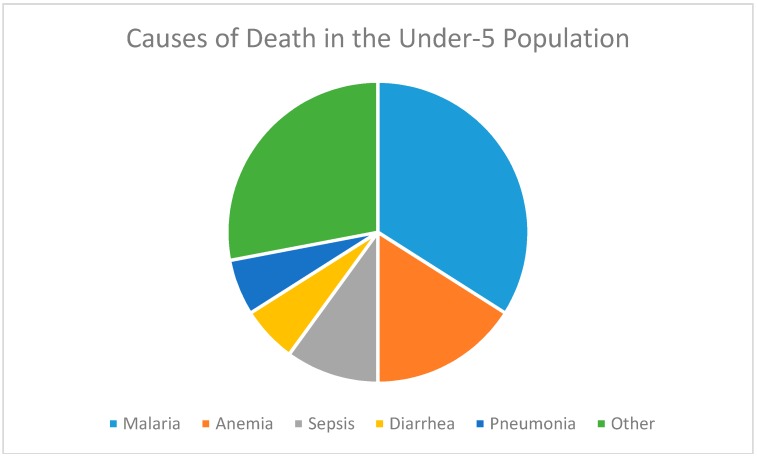
Distribution of diagnoses at death for children under five years of age.

Neonatal mortality in patients less than one month of age was most commonly secondary to sepsis, followed by tetanus and meningitis. One neonate was noted to have both tetanus and malaria. Deaths in patients one month to less than one year of age were most commonly due to malaria, followed by pneumonia. Similarly, the majority of pediatric deaths in patients at least one year of age to less than five years of age were due to malaria. The top five causes of pediatric mortality were malaria, anemia, sepsis, diarrhea, and pneumonia (Figure 1). Cases of pediatric death in patients five years of age and above were due to a variety of reasons, with no clear majority cause (Table 1).

When considering all pediatric deaths in those under five years old, there were 41 cases (82.0% of the total cases), with 51.2% of them attributed to malaria. The next five most common mortality causes were sepsis (12.2%), tetanus (7.3%), pneumonia (7.3%), diarrhea (7.3%), and meningitis (4.9%). Of the cases under five years old, nine cases (22.0%) were neonatal.

## 3. Discussion

Pediatric mortality at the Jackson F. Doe Regional Referral hospital (JFD) in 2013 was predominantly due to sepsis in the neonatal period, and malaria in the one month to under-five years of age group. The under-five age group comprised most of the pediatric deaths. The overall mortality rate was relatively low even in this setting without a pediatric intensive care unit. Despite limited resources, the mortality rate of 5.4% is comparable to the 6.4% described at Island Hospital in urban Monrovia in 2009 and lower than the 13.1% described at Mamba Point Hospital in 2005, one year post-war [4,5]. Multiple hospitals in Monrovia reported higher mortality rates of 11%, 14%, and 17% [5]. The higher mortality rates immediately post-war likely reflect the sequelae of the war’s effect on health infrastructure and overall child health. The mortality rate we report 10 years after the war may reflect a new baseline in the post-war, pre-Ebola era in Liberia, given the similarity to what was reported six years post-war [4].

This study found that the overall pediatric mortality rate at this hospital was also significantly lower than rates reported in other West African countries. Rates as high as 21% in a pediatric ward in Mali and 12% in a pediatric ward in Guinea-Bissau have been reported [6,7,8]. Notably, Guinea-Bissau saw an increase in pediatric mortality in the year following a war from 1998 to 1999, which was attributed to humanitarian aid and relief provisions present during the war that were not available post-war [9]. In contrast, Liberia saw an opposite trend, with higher pediatric mortality rates during the war compared to the immediate post-war period [2]. Our data corroborates this trend, with lower in-hospital mortality reported 10 years post-war compared to immediately post-war [4,5]. Additionally, the higher mortality rate in Nigeria may reflect more acutely ill patients presenting due to the presence of a pediatric intensive care unit, which was not available in our setting, or a better prehospital transport system, which allowed these patients to reach the hospital faster. Access to health care in Liberia is still an issue in rural settings, which affects health outcomes of the population [10].

Neonatal deaths are a significant proportion of pediatric mortality in under-resourced settings, particularly in West Africa. In Nigeria, as much as 48.4% of pediatric hospital deaths have been attributed to neonates, and several reports have shown birth asphyxia and prematurity to be more significant causes of neonatal death than sepsis [11]. In contrast, our data reveals neonatal deaths to comprise only 18.0% of the pediatric deaths, and sepsis is the leading cause. This discrepancy may reflect the accessibility of obstetric and neonatal care, or a relatively higher burden of infectious causes of death in our rural Liberia setting. Sepsis can also be a difficult diagnosis to exclude in neonates. Further exploration should be made to determine whether use of risk stratification criteria (i.e., Rochester criteria), consistent access to laboratory tests (i.e., serum tests, cerebrospinal fluid studies, cultures), and timely antibiotics are available. Additionally, in 2014 in Liberia, an overall estimated 35% of under-five-year-old deaths were neonatal, which may also suggest that a number of neonatal deaths are occurring out-of-hospital [12].

At JFD, the majority of pediatric deaths were in the under-five-year-old age group, and an overwhelming majority of these cases were due to infectious causes, particularly malaria, which comprised 51.2% of the cases. In Liberia, the diagnosis and treatment of malaria follows World Health Organization guidelines. JFD does have microscopy capacity for diagnosis and stocks anti-malarial medication. In 2009 in Liberia, the prevalence of malaria in children under five was 32%, and malaria has been reported as a leading cause of morbidity and mortality in the country [13]. Our data corroborates the known burden of malaria on child health in Liberia, and emphasizes that, especially in the cases where children are sick enough to be hospitalized, malaria has a high mortality rate. The hospital physicians recorded the cause of death, but children may have suffered from comorbidities such as malnutrition that affected outcomes, which was not documented.

Use of insecticide-treated bed nets (ITNs) has been shown to decrease the incidence of malaria and death due to malaria by 20% in several African countries [14]. In 2013, the World Bank–estimated percentage of insecticide-treated bed net use in Liberia was 38.1% while the United Nations Children's Fund (UNICEF) estimated 61.3% of households with at least one ITN and 20.1% of children sleeping under the ITNs [15,16]. Local studies show that there can be large discrepancies between the true local percentage and national reporting for a given site [17]. The actual usage of insecticide-treated bed nets may be even lower. Increasing the number of ITNs in the households and encouraging bed net use in the hospital wards may be a cost-effect measure to improve the burden of malaria and mortality secondary to malaria seen at JFD.

There were several cases of tetanus in our study. Tetanus can be a fatal disease that is preventable with vaccination. The coverage in Liberia has been improving steadily. In 2013, 91% of newborns in Liberia were protected from tetanus through maternal immunity, and 97% of children received the Diphtheria, Tetanus, and Pertussis 1 (DPT1) vaccine and 89% received the DPT3 vaccine [18,19]. In the previous year, 86% of the population received the DPT1 vaccine and 77% received DPT3 [16]. Two deaths in our study were in neonates, which is too early to receive the first tetanus vaccination. Practitioners should encourage all pregnant women to be fully vaccinated against tetanus to prevent neonatal tetanus. A common reason for neonates to acquire tetanus is when the umbilical cord is cut with an unsterilized instrument [18]. Administration of tetanus antitoxin can also be considered to counteract the infection.

While our data is limited due to small sample size and the limitations of record-keeping in an under-resourced setting, our study may provide support for developing strategies to continue the improvement of child health in Liberia. This hospital is the main tertiary hospital in northern Liberia and the results in this study could provide an estimation of the child mortality causes with obvious limitations. Our work demonstrates the need for focused efforts on decreasing neonatal sepsis, tetanus vaccination, as well as malaria prevention. The importance of addressing neonatal sepsis has been established on a global scale, and our data emphasizes that Liberia is no exception. In a large multi-center study analyzing trends among the causes of childhood mortality worldwide, neonatal sepsis was one of the causes that has seen the slowest progress [20].

With regards to addressing childhood mortality due to malaria in Liberia, our data supports the need for ongoing efforts in both community health and inpatient treatment. A hospital in Guinea-Bissau was able to show that training hospital personnel in standardized guidelines for malaria treatment reduced in-hospital mortality in children under five years of age [21]. Additionally, community management of malaria has improved infant mortality [22]. Therefore, these may be avenues in which focused efforts have the potential to result in a measurable impact on the child health of Liberia as it looks toward achieving the sustainable development goals set forth by the United Nations for 2030 to continue driving down neonatal under-five mortality.

## 4. Materials and Methods

### 4.1. Study Setting

The Jackson F. Doe Regional Referral hospital (JFD) is a rural tertiary care hospital in Tappita, Liberia. The hospital catchment area includes neighboring countries Guinea and Ivory Coast. While some patients of lower socioeconomic status may not be able to afford the travel to this facility, the majority of the patient population is of low socioeconomic status from the area around the hospital. People who have the economic means sometimes will come here for care from the capital city since this hospital has the only computed tomography scanner in the country.

The hospital has a pediatric ward with forty beds, four beds for critically ill patients, but no intensive care unit. Oxygen, pulse oximetry, and blood pressure monitoring is available, but there are no ventilators, telemetry monitoring, or electrocardiograms. Oxygen concentrators are supplied from the capital city, a nine hour drive away, but dependent on possible transportation issues. At any given time, the pediatric ward is staffed with one to two nurses, one pediatrician, and up to two rotating interns.

### 4.2. Study Design

This is a one-year retrospective review of pediatric mortality at a rural tertiary care hospital in northern Liberia between January 2013 and December 2013. Any patient under the age of eighteen who had deaths recorded in the patient logbooks were included. No patients were excluded from analysis. JFD patient paper log books were reviewed and data was transferred to Microsoft Excel sheets. The mortality diagnosis was based on patient history, physical examination, laboratory tests, and imaging tests. Patients were diagnosed by the pediatrician on the ward and documented in the medical record books.

The main causes of death were analyzed. Age groups were subdivided into neonates, one month old to under 12 months, one year old to under five years old, and five years old and above. The major causes of death were described for each age group. Because the under-five mortality rate is a leading indicator of child health in countries, the causes of mortality of all children under the age of five will be described.

The University of Texas at Southwestern Medical Center Institutional Review Board approved this study for exemption.

## 5. Conclusions

Pediatric mortality in the under-five-year-old group at Jackson F. Doe hospital in northern Liberia is still mostly due to infectious disease, particularly malaria. Continuing efforts should be dedicated to the prevention and treatment of malaria. Further pediatric mortality monitoring and evaluation should continue to evaluate for changes in trends.

## Figures and Tables

**Table 1:** Causes of pediatric death stratified by age group.

	Number of Deaths (% of Total Deaths in Age Group)				
Cause of Death	Neonates (<1 Month) *N* = 9	1 Month to <1 Year, *N* = 9	1 Year to <5 Year, *N* = 23	≥5 Years, *N* = 9	Total
Sepsis	4	1	0	0	5
Tetanus	2 *	1	0	1	4
Meningitis	2	0	0	0	2
Pneumothorax	1	0	0	0	1
Malaria	1 *	4	16	2	23
Pneumonia	0	2	1	1	4
Diarrhea	0	1	2	1	4
Caustic ingestion	0	0	1	0	1
Malnutrition	0	0	1	0	1
Perioperative complications	0	0	1	0	1
Anemia	0	0	1	1	2
Acute abdomen	0	0	0	1	1
Congestive heart failure	0	0	0	1	1
Abdominal mass	0	0	0	1	1

* One neonate’s death was attributed to both tetanus and malaria.
